# Supplementary material for: Clinical test cases for commissioning, QA, and benchmarking of model‐based dose calculation algorithms in 1⁹2Ir HDR gynecologic tandem and ring brachytherapy
Source: Med Phys. 2026 Jan 8;53(1):e70260. doi: 10.1002/mp.70260 (PMC12783015; doi:10.1002/mp.70260)
Supplement: Supplementary file 1 — Supporting information [file MP-53-0-s001.pdf]

TABLE S1. Dwell positions, direction cosines and dwell time weights used in the MCNP, RapidBrachyMCTPS and eb_gui simulations for test cases A and B.

	**Dwell Positions**			**Direction Cosines**			**Dwell Time Weights**
	**x (cm)**	**y (cm)**	**z (cm)**	**cosx**	**cosy**	**cosz**	
**Catheter 1**	-5.2422	-20.0126	-2.1424	0.915153	-0.129507	-0.381719	0.04167
	-5.6686	-19.9177	-1.9123	0.738578	-0.300268	-0.603601	0.04167
	-5.9616	-19.7291	-1.5673	0.425680	-0.457597	-0.780424	0.04167
	-6.0974	-19.4822	-1.1629	0.111006	-0.531614	-0.838783	0.04167
	-6.1079	-19.2084	-0.7449	0.006869	-0.549305	-0.835539	0.04167
	-6.0177	-18.9296	-0.3436	-0.243756	-0.560568	-0.791411	0.04167
	-2.9910	-18.6026	-0.4722	-0.332303	0.486177	0.807869	0.04167
	-2.8330	-18.8480	-0.8780	-0.302121	0.494824	0.814735	0.04167
	-2.7726	-19.1105	-1.2872	0.093662	0.560395	0.822853	0.04167
	-2.8524	-19.3926	-1.6915	0.190711	0.566083	0.801919	0.04167
	-3.0964	-19.6484	-2.0284	0.615281	0.488253	0.618878	0.04167
	-3.4834	-19.8467	-2.2649	0.973311	-0.097823	0.207594	0.04167
**Catheter 2**	-3.8115	-23.9380	2.3312	0.113416	-0.829494	0.546878	0.04167
	-3.8682	-23.5233	2.0577	0.113416	-0.829494	0.546878	0.04167
	-3.9249	-23.1085	1.7843	0.113416	-0.829494	0.546878	0.04167
	-3.9816	-22.6938	1.5108	0.113416	-0.829494	0.546878	0.04167
	-4.0383	-22.2790	1.2374	0.113416	-0.829494	0.546878	0.04167
	-4.0951	-21.8643	0.9640	0.113416	-0.829494	0.546878	0.04167
	-4.1518	-21.4495	0.6905	0.113416	-0.829494	0.546878	0.04167
	-4.2085	-21.0348	0.4171	0.113416	-0.829494	0.546878	0.04167
	-4.2652	-20.6200	0.1436	0.113416	-0.829494	0.546878	0.04167
	-4.3219	-20.2053	-0.1298	0.113416	-0.829494	0.546878	0.04167
	-4.3786	-19.7906	-0.4032	0.113416	-0.829494	0.546878	0.04167
	-4.4405	-19.3626	-0.6534	0.129449	-0.870355	0.475095	0.04167

TABLE S2. CT calibration of BracyVision TPS used to convert HUs to mass densities in the MCNP, RapidBrachyMCTPS and eb_gui simulations for test cases A and B.

HU	Density (g/cm^3)
-1000	0.00111
-992	0.00121
-976	0.00131
-480	0.50000
-96	0.95000
0	1.00000
48	1.05000
128	1.10000
528	1.33400
976	1.60300
1488	1.85000
1824	2.10000
2224	2.40000
2640	2.70000
2832	2.83000
3500	3.55500

TABLE S3. Elemental composition assigned to the scoring voxels of test cases A and B in the MCNP, RapidBrachyMCTPS and eb_gui simulations.

	% mass												Density (g/cm^3)
	**H**	**C**	**N**	**O**	**Na**	**Mg**	**P**	**S**	**Cl**	**Ar**	**K**	**Ca**	
**Air**	-	-	75.5	23.2	-	-	-	-	-	1.3	-	-	[0, 0.08)
**Lung**	10.3	10.5	3.1	74.9	0.2	-	0.2	0.3	0.3	-	0.2	-	[0.08, 0.907)
**Soft Tissues**	11.6	68.1	0.2	19.8	0.1	-	-	0.1	0.1	-	-	-	[0.907, 0.945)
	11.3	56.7	0.9	30.8	0.1	-	-	0.1	0.1	-	-	-	[0.945, 0.971)
	11.0	45.8	1.5	41.1	0.1	-	0.1	0.2	0.2	-	-	-	[0.971, 0.997)
	10.8	35.6	2.2	50.9	-	-	0.1	0.2	0.2	-	-	-	[0.997, 1.024)
	10.6	28.4	2.6	57.8	-	-	0.1	0.2	0.2	-	0.1	-	[1.024, 1.030)
	9.9	17.1	4.6	67.2	0.4	-	0.1	0.4	0.3	-	0.1	-	[1.030, 1.088)
**Skeletal Tissues**	9.5	45.5	2.5	35.5	0.1	-	2.1	0.1	0.1	-	0.1	4.5	[1.088, 1.135)
	8.9	42.3	2.7	36.3	0.1	-	3.0	0.1	0.1	-	0.1	6.4	[1.135, 1.195)
	8.2	39.1	2.9	37.2	0.1	-	3.9	0.1	0.1	-	0.1	8.3	[1.195, 1.254)
	7.6	36.1	3.0	38.0	0.1	0.1	4.7	0.2	0.1	-	-	10.1	[1.254, 1.313)
	7.1	33.5	3.2	38.7	0.1	0.1	5.4	0.2	-	-	-	11.7	[1.313, 1.372)
	6.6	31.0	3.3	39.4	0.1	0.1	6.1	0.2	-	-	-	13.2	[1.372, 1.431)
	6.1	28.7	3.5	40.0	0.1	0.1	6.7	0.2	-	-	-	14.6	[1.431, 1.491)
	5.6	26.5	3.6	40.5	0.1	0.2	7.3	0.3	-	-	-	15.9	[1.491, 1.550)
	5.2	24.6	3.7	41.1	0.1	0.2	7.8	0.3	-	-	-	17.0	[1.550, 1.609)
	4.9	22.7	3.8	41.6	0.1	0.2	8.3	0.3	-	-	-	18.1	[1.609, 1.668)
	4.5	21.0	3.9	42.0	0.1	0.2	8.8	0.3	-	-	-	19.2	[1.668, 1.727)
	4.2	19.4	4.0	42.5	0.1	0.2	9.2	0.3	-	-	-	20.1	[1.727, 1.787)
	3.9	17.9	4.1	42.9	0.1	0.2	9.6	0.3	-	-	-	21.0	[1.787, 1.846)
	3.6	16.5	4.2	43.2	0.1	0.2	10.0	0.3	-	-	-	21.9	[1.846, 1.905)
	3.4	15.5	4.2	43.5	0.1	0.2	10.3	0.3	-	-	-	22.5	[1.905, 1.964)
**PPSU**	5.0	73.3	-	14.5	-	-	-	7.2	-	-	-	-	1.2951

TABLE S4. Comparison of target and critical organs-related dosimetry for test case A in the form of different indices obtained from MCNP, RapidBrachyMCTPS and eb_gui based DVHs calculated independently from the TPSs.

		Dose data used for DVH calculation			$\%\Delta D_{LOCAL}$[a]	
ROI	DVH indices	(a) MCNP	(b) RapidBrachy MCTPS	(c) eb_gui	(b – a)	(c - a)
Target	D50 (Gy)	9.07	9.06	9.03	0.46	0.36
	D90 (Gy)	5.54	5.54	5.52	-0.05	-0.35
	D98 (Gy)	4.68	4.67	4.66	0.39	0.19
Bladder	D0.1cc (Gy)	7.16	7.14	7.13	-0.18	-0.35
	D2cc (Gy)	5.75	5.72	5.73	-0.52	-0.44
	D50 (Gy)	1.74	1.73	1.73	0.48	-0.03
	D98 (Gy)	0.87	0.86	0.86	0.50	-0.06
Bowel	D0.1cc (Gy)	5.43	5.40	5.41	-0.55	-0.35
	D2cc (Gy)	3.71	3.69	3.69	-0.58	-0.51
	D50 (Gy)	0.82	0.81	0.81	0.46	-0.12
	D98 (Gy)	0.26	0.26	0.26	0.36	-0.28
Rectum	D0.1cc (Gy)	2.32	2.31	2.31	-0.31	-0.42
	D2cc (Gy)	1.97	1.97	1.96	-0.23	-0.46
	D50 (Gy)	0.89	0.88	0.88	0.44	0.13
	D98 (Gy)	0.49	0.48	0.48	0.48	-0.13
Sigmoid	D0.1cc (Gy)	4.08	4.06	4.06	-0.56	-0.49
	D2cc (Gy)	3.21	3.19	3.20	-0.55	-0.38
	D50 (Gy)	1.31	1.30	1.30	0.48	-0.15
	D98 (Gy)	0.35	0.35	0.35	0.40	-0.09
Bones	D0.1cc (Gy)	1.42	1.41	1.41	-0.56	-0.41
	D2cc (Gy)	1.13	1.13	1.13	-0.54	-0.57
	D50 (Gy)	0.29	0.29	0.29	0.58	0.08
	D98 (Gy)	0.03	0.03	0.03	0.68	0.00

[a] $\Delta D_{LOCAL} = 100 \times \{(b\ or\ c)/(a) - 1\}$

TABLE S5. Comparison of target and critical organs-related dosimetry for test case B in the form of different indices obtained from MCNP, RapidBrachyMCTPS and eb_gui based DVHs calculated independently from the TPSs.

		**Dose data used for DVH calculation**			**$\%\Delta D_{LOCAL}$[b]**	
**ROI**	**DVH indices**	**(a) MCNP**	**(b) RapidBrachy MCTPS**	**(c) eb_gui**	**(b – a)**	**(c - a)**
**Target**	D50 (%)	9.07	9.00	9.02	0.49	-0.20
	D90 (Gy)	5.54	5.51	5.52	-0.59	-0.46
	D98 (Gy)	4.68	4.65	4.66	0.53	-0.11
**Bladder**	D0.1cc (Gy)	7.13	7.13	7.10	-0.07	-0.49
	D2cc (Gy)	5.74	5.71	5.71	-0.53	-0.52
	D50 (Gy)	1.73	1.72	1.72	0.50	-0.01
	D98 (Gy)	0.86	0.86	0.86	0.38	-0.15
**Bowel**	D0.1cc (Gy)	5.38	5.34	5.34	-0.81	-0.74
	D2cc (Gy)	3.68	3.65	3.65	-0.61	-0.62
	D50 (Gy)	0.81	0.81	0.81	0.55	0.18
	D98 (Gy)	0.26	0.26	0.26	0.71	0.51
**Rectum**	D0.1cc (Gy)	2.29	2.28	2.29	-0.47	-0.02
	D2cc (Gy)	1.93	1.92	1.92	-0.63	-0.23
	D50 (Gy)	0.87	0.86	0.87	0.40	-0.21
	D98 (Gy)	0.51	0.51	0.51	0.47	-0.17
**Sigmoid**	D0.1cc (Gy)	4.05	4.02	4.02	-0.76	-0.73
	D2cc (Gy)	3.18	3.17	3.17	-0.52	-0.44
	D50 (Gy)	1.30	1.30	1.30	0.44	0.06
	D98 (Gy)	0.36	0.36	0.36	0.37	0.37
**Right FH**	D0.1cc (Gy)	0.71	0.70	0.70	-0.90	-1.09
	D2cc (Gy)	0.61	0.60	0.60	-0.77	-0.91
	D50 (Gy)	0.39	0.39	0.39	0.65	-0.08
	D98 (Gy)	0.24	0.24	0.24	0.13	-0.71
**Left FH**	D0.1cc (Gy)	0.65	0.64	0.64	-0.92	-0.96
	D2cc (Gy)	0.56	0.56	0.56	-0.51	-0.60
	D50 (Gy)	0.36	0.35	0.36	0.36	0.35
	D98 (Gy)	0.22	0.22	0.22	0.22	0.22
**Marrow**	D0.1cc (Gy)	1.02	1.01	1.01	-1.05	-1.08
	D2cc (Gy)	0.90	0.89	0.89	-0.63	-0.69
	D50 (Gy)	0.43	0.43	0.43	0.40	-0.18
	D98 (Gy)	0.12	0.11	0.12	0.17	-0.10
**Pelvic Bones**	D0.1cc (Gy)	1.40	1.39	1.39	-0.86	-0.64
	D2cc (Gy)	1.23	1.22	1.23	-0.76	-0.65
	D50 (Gy)	0.41	0.41	0.41	0.46	-0.11
	D98 (Gy)	0.12	0.12	0.12	0.34	-0.06

[b] $\Delta D_{LOCAL} = 100 \times \{(b,c)/(a) - 1\}$

TABLE S6. Comparison of target and critical organs-related dosimetry for test case A in the form of different indices obtained from MCNP, ACE (High Accuracy), ACE (Standard Accuracy) and TG-43 based DVHs calculated using OncentraBrachy TPS.

		$DVH_{MC,ref}$	$DVH_{TPS,ref}$			$\%\Delta D_{LOCAL}$[c]		
**ROI**	**DVH indices**	**(a) MCNP**	**(b) ACE (HA)**	**(c) ACE (SA)**	**(d) TG-43**	**(b – a)**	**(c – a)**	**(d - a)**
**Target**	D50 (Gy)	9.51	9.56	9.59	9.67	0.56	0.89	1.67
	D90 (Gy)	5.70	5.72	5.75	5.81	0.40	0.95	1.99
	D98 (Gy)	4.82	4.84	4.87	4.92	0.27	1.00	1.99
**Bladder**	D0.1cc (Gy)	6.92	6.99	7.02	7.02	1.09	1.45	1.42
	D2cc (Gy)	5.66	5.72	5.74	5.74	0.93	1.41	1.40
	D50 (Gy)	1.74	1.76	1.76	1.79	1.01	1.26	2.99
	D98 (Gy)	0.87	0.89	0.89	0.92	1.54	1.37	5.55
**Bowel**	D0.1cc (Gy)	5.13	5.11	5.14	5.19	-0.41	0.03	1.10
	D2cc (Gy)	3.62	3.61	3.64	3.68	-0.36	0.68	1.56
	D50 (Gy)	0.88	0.84	0.85	0.85	-4.26	-3.30	-3.01
	D98 (Gy)	0.26	0.28	0.28	0.29	4.76	4.95	12.19
**Rectum**	D0.1cc (Gy)	2.28	2.29	2.29	2.36	0.44	0.48	3.64
	D2cc (Gy)	1.93	1.94	1.94	2.01	0.39	0.08	3.72
	D50 (Gy)	0.89	0.90	0.90	0.94	0.67	0.45	5.32
	D98 (Gy)	0.50	0.51	0.50	0.54	1.71	1.61	8.16
**Sigmoid**	D0.1cc (Gy)	4.02	4.00	4.05	4.08	-0.31	0.78	1.49
	D2cc (Gy)	3.15	3.15	3.18	3.21	-0.22	0.86	1.76
	D50 (Gy)	1.32	1.35	1.31	1.32	2.97	-0.23	0.11
	D98 (Gy)	0.36	0.37	0.37	0.39	2.67	2.81	8.57
**Bones**	D0.1cc (Gy)	1.17	1.08	1.08	1.09	-8.04	-7.74	-6.80
	D2cc (Gy)	0.95	0.87	0.87	0.89	-8.50	-8.56	-6.98
	D50 (Gy)	0.20	0.18	0.18	0.19	-11.39	-12.87	-4.46
	D98 (Gy)	0.03	0.03	0.03	0.03	17.86	8.93	3.57

[c] $\%\Delta D_{LOCAL} = 100 \times \{(b, c, d)/(a) - 1\}$

TABLE S7. Comparison of target and critical organs-related dosimetry for test case B in the form of different indices obtained from MCNP, ACE (High Accuracy), ACE (Standard Accuracy) and TG-43 based DVHs calculated using OncentraBrachy TPS.

		$DVH_{MC,ref}$	$DVH_{TPS,ref}$			$\%\Delta D_{LOCAL}$[d]		
**ROI**	**DVH indices**	**(a) MCNP**	**(b) ACE (HA)**	**(c) ACE (SA)**	**(d) TG-43**	**(b – a)**	**(c – a)**	**(d - a)**
**Target**	D50 (Gy)	9.51	9.54	9.57	9.67	0.38	0.69	1.69
	D90 (Gy)	5.69	5.70	5.73	5.81	0.16	0.73	2.06
	D98 (Gy)	4.82	4.83	4.86	4.92	0.02	0.73	1.97
**Bladder**	D0.1cc (Gy)	6.90	6.97	7.00	7.02	1.08	1.47	1.77
	D2cc (Gy)	5.64	5.70	5.73	5.74	1.04	1.59	1.85
	D50 (Gy)	1.73	1.75	1.75	1.79	1.04	1.18	3.34
	D98 (Gy)	0.87	0.88	0.88	0.92	1.50	1.21	6.34
**Bowel**	D0.1cc (Gy)	5.08	5.07	5.11	5.19	-0.18	0.49	2.17
	D2cc (Gy)	3.58	3.58	3.62	3.68	-0.01	1.01	2.64
	D50 (Gy)	0.84	0.84	0.84	0.88	0.66	0.84	5.07
	D98 (Gy)	0.26	0.28	0.28	0.29	4.36	4.55	11.76
**Rectum**	D0.1cc (Gy)	2.25	2.30	2.30	2.36	2.31	2.40	5.00
	D2cc (Gy)	1.90	1.95	1.95	2.01	2.77	2.53	5.66
	D50 (Gy)	0.88	0.91	0.91	0.94	3.87	3.64	6.99
	D98 (Gy)	0.52	0.54	0.54	0.54	3.72	3.53	2.48
**Sigmoid**	D0.1cc (Gy)	3.99	3.98	4.03	4.07	-0.23	0.99	2.23
	D2cc (Gy)	3.12	3.14	3.17	3.21	0.45	1.58	2.74
	D50 (Gy)	1.31	1.31	1.31	1.35	0.27	0.04	3.68
	D98 (Gy)	0.36	0.37	0.37	0.39	2.34	2.34	6.47
**Right FH**	D0.1cc (Gy)	0.70	0.66	0.66	0.68	-5.61	-5.54	-2.52
	D2cc (Gy)	0.61	0.57	0.57	0.60	-5.12	-5.29	-0.83
	D50 (Gy)	0.39	0.37	0.37	0.40	-4.11	-4.11	2.44
	D98 (Gy)	0.24	0.24	0.24	0.27	-0.62	-1.03	10.72
**Left FH**	D0.1cc (Gy)	0.64	0.60	0.60	0.62	-6.13	-5.89	-2.91
	D2cc (Gy)	0.56	0.52	0.52	0.55	-5.93	-6.11	-1.62
	D50 (Gy)	0.36	0.34	0.34	0.37	-4.64	-4.92	2.67
	D98 (Gy)	0.22	0.22	0.22	0.25	-0.67	0.00	11.19
**Marrow**	D0.1cc (Gy)	1.03	0.96	0.97	0.99	-5.90	-5.51	-3.22
	D2cc (Gy)	0.89	0.88	0.88	0.91	-1.74	-1.46	1.80
	D50 (Gy)	0.43	0.42	0.42	0.44	-2.22	-2.10	3.38
	D98 (Gy)	0.12	0.12	0.12	0.14	0.85	1.70	21.28
**Pelvic Bones**	D0.1cc (Gy)	1.37	1.33	1.33	1.35	-2.34	-2.34	-1.35
	D2cc (Gy)	1.22	1.19	1.20	1.20	-1.85	-1.77	-1.07
	D50 (Gy)	0.41	0.40	0.40	0.42	-1.47	-1.83	2.32
	D98 (Gy)	0.12	0.12	0.12	0.14	-0.84	-2.10	13.87

[d] $\%\Delta D_{LOCAL} = 100 \times \{(b,c,d)/(a) - 1\}$

TABLE S8. Comparison of target and critical organs-related dosimetry for test case A in the form of different indices obtained from MCNP, Acuros BV and TG-43 based DVHs calculated using BrachyVision TPS.

		$DVH_{MC,ref}$	$DVH_{TPS,ref}$		$\%\Delta D_{LOCAL}$[e]	
**ROI**	**DVH indices**	**(a) MCNP**	**(b) Acuros BV**	**(c) TG43**	**(b – a)**	**(c - a)**
**Target**	D50 (Gy)	9.53	9.51	9.68	-0.16	1.57
	D90 (Gy)	5.70	5.70	5.82	0.09	2.19
	D98 (Gy)	4.83	4.83	4.94	0.10	2.38
**Bladder**	D0.1cc (Gy)	6.96	6.94	7.04	-0.29	1.15
	D2cc (Gy)	5.71	5.69	5.78	-0.26	1.23
	D50 (Gy)	1.75	1.76	1.80	0.57	3.15
	D98 (Gy)	0.88	0.88	0.93	0.57	6.29
**Bowel**	D0.1cc (Gy)	5.18	5.18	5.24	0.00	1.16
	D2cc (Gy)	3.65	3.65	3.70	0.14	1.51
	D50 (Gy)	0.83	0.83	0.87	0.61	5.45
	D98 (Gy)	0.27	0.27	0.30	0.00	13.21
**Rectum**	D0.1cc (Gy)	2.29	2.30	2.37	0.22	3.49
	D2cc (Gy)	1.95	1.95	2.02	0.26	3.60
	D50 (Gy)	0.89	0.90	0.94	0.56	5.62
	D98 (Gy)	0.50	0.50	0.54	0.00	8.08
**Sigmoid**	D0.1cc (Gy)	4.04	4.04	4.10	-0.12	1.49
	D2cc (Gy)	3.18	3.18	3.24	0.16	2.05
	D50 (Gy)	1.31	1.32	1.36	0.38	3.44
	D98 (Gy)	0.36	0.36	0.39	0.00	9.86
**Bones**	D0.1cc (Gy)	1.24	1.23	1.18	-1.21	-5.24
	D2cc (Gy)	1.06	1.03	0.99	-2.83	-6.60
	D50 (Gy)	0.23	0.22	0.23	-4.35	-2.17
	D98 (Gy)	0.03	0.03	0.06	0.00	140.00

[e] $\%\Delta D_{LOCAL} = 100 \times \{(b, c, d)/(a) - 1\}$

TABLE S9. Comparison of target and critical organs-related dosimetry for test case B in the form of different indices obtained from MCNP, Acuros BV and TG-43 based DVHs calculated using BrachyVision TPS.

		$DVH_{MC,ref}$	$DVH_{TPS,ref}$		$\%\Delta D_{LOCAL}$[f]	
**ROI**	**DVH indices**	**(a) MCNP**	**(b) Acuros BV**	**(c) TG43**	**(b – a)**	**(c – a)**
**Target**	D50 (Gy)	9.52	9.49	9.68	-0.26	1.68
	D90 (Gy)	5.69	5.69	5.82	-0.09	2.28
	D98 (Gy)	4.83	4.83	4.94	0.00	2.38
**Bladder**	D0.1cc (Gy)	6.93	6.92	7.04	-0.14	1.59
	D2cc (Gy)	5.69	5.68	5.78	-0.18	1.58
	D50 (Gy)	1.74	1.76	1.80	0.86	3.45
	D98 (Gy)	0.87	0.88	0.93	1.15	6.90
**Bowel**	D0.1cc (Gy)	5.12	5.12	5.24	0.00	2.25
	D2cc (Gy)	3.61	3.63	3.70	0.42	2.49
	D50 (Gy)	0.82	0.83	0.87	1.22	6.10
	D98 (Gy)	0.27	0.27	0.30	1.89	13.21
**Rectum**	D0.1cc (Gy)	2.26	2.32	2.37	2.43	4.65
	D2cc (Gy)	1.91	1.97	2.02	3.14	5.50
	D50 (Gy)	0.88	0.93	0.94	5.71	7.43
	D98 (Gy)	0.52	0.55	0.54	5.77	2.88
**Sigmoid**	D0.1cc (Gy)	4.01	4.01	4.10	0.00	2.37
	D2cc (Gy)	3.15	3.17	3.25	0.79	3.18
	D50 (Gy)	1.31	1.31	1.36	0.38	3.83
	D98 (Gy)	0.36	0.37	0.39	1.39	8.33
**Right FH**	D0.1cc (Gy)	0.70	0.68	0.68	-2.86	-2.86
	D2cc (Gy)	0.61	0.60	0.61	-2.46	-0.82
	D50 (Gy)	0.39	0.39	0.40	-1.28	2.56
	D98 (Gy)	0.25	0.25	0.28	0.00	12.24
**Left FH**	D0.1cc (Gy)	0.64	0.62	0.62	-3.13	-3.13
	D2cc (Gy)	0.56	0.55	0.55	-1.79	-1.79
	D50 (Gy)	0.36	0.35	0.37	-1.41	2.82
	D98 (Gy)	0.23	0.23	0.26	0.00	13.33
**Marrow**	D0.1cc (Gy)	1.03	1.02	1.00	-0.97	-2.91
	D2cc (Gy)	0.90	0.90	0.92	0.56	2.23
	D50 (Gy)	0.43	0.43	0.45	0.00	3.49
	D98 (Gy)	0.12	0.12	0.16	0.00	34.78
**Pelvic Bones**	D0.1cc (Gy)	1.37	1.35	1.36	-1.82	-0.73
	D2cc (Gy)	1.23	1.21	1.22	-1.63	-0.82
	D50 (Gy)	0.41	0.41	0.42	0.00	2.44
	D98 (Gy)	0.12	0.12	0.15	0.00	25.00

[f] $\%\Delta D_{LOCAL} = 100 \times \{(b,c,d)/(a) - 1\}$
